# Cardiac arrest due to lymphocytic colitis: a case report

**DOI:** 10.1186/1752-1947-6-80

**Published:** 2012-03-09

**Authors:** Kristian A Groth, Jens Kelsen, Bo Løfgren

**Affiliations:** 1 Department of Cardiology, Aarhus University Hospital, Skejby, Brendstrupgaardsvej 100, DK-8200 Aarhus N, Denmark; 2 Department of Medicine, Randers Regional Hospital, Randers, Skovlyvej 1, DK-8930 Randers, Denmark; 3 Research Center for Emergency Medicine, Aarhus University Hospital, Aarhus Sygehus, Nørrebrogade 44, DK 8000 Aarhus C, Denmark

## Abstract

**Introduction:**

We present a case of cardiac arrest due to hypokalemia caused by lymphocytic colitis.

**Case presentation:**

A 69-year-old Caucasian man presented four months prior to a cardiac arrest with watery diarrhea and was diagnosed with lymphocytic colitis. Our patient experienced a witnessed cardiac arrest at his general practitioner's surgery. Two physicians and the emergency medical services resuscitated our patient for one hour and four minutes before arriving at our university hospital. Our patient was defibrillated 16 times due to the recurrence of ventricular tachyarrhythmias. An arterial blood sample revealed a potassium level of 2.0 mmol/L (reference range: 3.5 to 4.6 mmol/L) and pH 6.86 (reference range: pH 7.37 to 7.45). As the potassium level was corrected, the propensity for ventricular tachyarrhythmias ceased. Our patient recovered from his cardiac arrest without any neurological deficit. Further tests and examinations revealed no other reason for the cardiac arrest.

**Conclusion:**

Diarrhea can cause life-threatening situations due to the excretion of potassium, ultimately causing cardiac arrest due to hypokalemia. Physicians treating patients with severe diarrhea should consider monitoring their electrolyte levels.

## Introduction

Untreated inflammatory colitis normally presents with chronic watery diarrhea. Potassium is secreted in the colon and is eliminated in diarrhea; therefore, profuse diarrhea can result in hypokalemia. The myocardium is extremely sensitive to hypokalemia and alters cardiac tissue excitability and conduction, which may induce malignant ventricular arrhythmias, resulting in cardiac arrest.

## Case presentation

A 69-year-old Caucasian man experienced a witnessed cardiac arrest at his general practitioner's surgery. Two physicians immediately initiated cardiopulmonary resuscitation (CPR), that is chest compression and mouth-to-mouth ventilation. The emergency medical services were summoned and an ambulance arrived eight minutes later. The initial cardiac rhythm was ventricular fibrillation. Our patient was defibrillated 10 times. Our patient gained an organized rhythm several times post-defibrillation. He was treated with amiodarone, adrenaline, atropine and lidocaine. One hour and four minutes after the first heart rhythm analysis by the emergency medical technician, our patient arrived at our university hospital still in cardiac arrest.

Upon arrival, mechanical chest compressions were started using the LUCAS™ 2 Chest Compression System (Physio-Control Inc./Jolife AB, SE-223 70 Lund, Sweden) and our patient was defibrillated twice. A coronary angiography did not show any signs of coronary artery disease. Echocardiography also did not reveal any signs of structural cardiac disease. A biochemical analysis showed a potassium level of 2.0 mmol/L (reference range: 3.5 to 4.6 mmol/L) and pH 6.86 (reference range: pH 7.37 to 7.45).

Our patient was admitted to our intensive care unit (ICU) and was defibrillated a further four times due to the recurrence of ventricular tachyarrhythmias. As his serum potassium level was corrected, the propensity for ventricular tachyarrhythmias ceased. After 24 hours in the ICU our patient was transferred to the cardiac care ward, where no arrhythmias were observed during the following 24 hours. Subsequently, our patient was transferred to our department of gastroenterology. He was assessed by a neurologist three months after the cardiac arrest and found to be neurologically intact.

**Figure 1:**
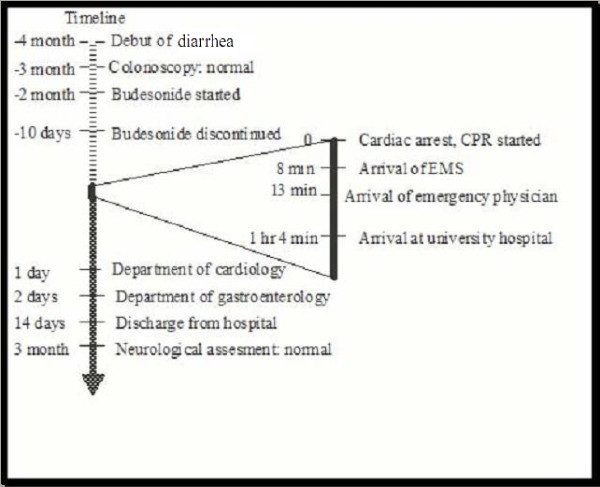
**Timeline divided in three parts**. Diagnostic phase prior to the cardiac arrest, cardiac arrest phase and rehabilitation phase after the cardiac arrest. CPR: Cardiopulmonary Resuscitation, EMS: Emergency Medical Service.

Four months prior to the cardiac arrest (Figure 1), our patient had presented with chronic watery diarrhea. He initially reported continuous diarrhea with four stools per day without warning symptoms. A colonoscopy showed his colon to have a normal macroscopic appearance. Histological evaluation revealed lymphocytic colitis and our patient started treatment with budesonide. At the time of diagnosis his only reported medication was acetylsalicylic acid. Our patient was not recommended to stop his acetylsalicylic acid intake due to a previous history of stroke.

Ten days before experiencing the cardiac arrest, our patient had consulted the gastroenterology outpatient clinic due to the lack of effect of the budesonide. The glucocorticoid was discontinued and our patient was recommended psyllium seed treatment, from which he had previously experienced some relief. His potassium level was found to be normal three times during his first two and a half months with diarrhea, but was not monitored during the last one and a half months prior to the cardiac arrest.

## Discussion

Microscopic colitis is characterized by a (near) normal colonoscopy. The diagnosis is established by biopsy of the colonic mucosa. The severity of histological change is most prominent in the proximal colon and declines distally; that is, biopsies from the right or transverse colon are optimal. On histopathology, the condition is separate from collagenous colitis and lymphocytic colitis [1]. The etiology is multifactorial and it is unknown whether the two subtypes are pathogenetically related [2]. The peak incidence of the disease is in the age range of 55 to 70 years and occurs predominantly in women (female to male ratio, 2 to 3:1) [3]. Although most cases are idiopathic, certain drugs can induce the condition, particularly nonsteroidal anti-inflammatory drugs but also acetylsalicylic acid [3]. Other drugs which can induce colitis include acarbose, ranitidine, sertraline, ticlopidine, flutamide, omeprazole and simvastatin [4].

Microscopic colitis may be diagnosed in 10% to 20% of cases investigated for chronic watery diarrhea [2] of up to two liters per day (four to nine stools daily; sometimes > 10 stools daily). The mechanism of diarrhea in collagenous colitis is believed to be a decreased absorption and increased secretion of sodium and chloride ions [5]. The absorptive mechanisms of potassium ions are not disturbed by diarrhea *per se*, but fecal potassium losses are increased in diarrheal diseases by unabsorbed anions (which obligate potassium loss), by electrochemical gradients secondary to active chloride secretion and probably by secondary hyperaldosteronism [6].

When the diagnosis of lymphocytic colitis has been confirmed, the patient's use of drugs and dietary factors that may contribute to diarrhea must be evaluated. Drugs associated with lymphocytic colitis should be stopped. Excessive intake of dairy products, caffeine and alcohol should be reduced. Celiac disease and bile acid malabsorption should be excluded. If symptoms are debilitating, budesonide, a glucocorticoid with low systemic effect due to its substantial elimination by first-pass hepatic metabolism, has been proven effective [7]. The long-term prognosis for patients suffering from lymphocytic colitis may be more favorable compared to collagenous colitis; diarrhea may subside within weeks with or without treatment and histologic findings may normalize.

In our case, our patient suffered from cardiac arrest due to hypokalemia. The hypokalemia was caused by diarrhea as a consequence of lymphocytic colitis. One case of hypokalemia-induced electrocardiogram changes due to microscopic colitis has previously been reported [8]. Hypokalemia is a known side effect of budesonide treatment. Our patient stopped budesonide treatment ten days before his cardiac arrest. After his cardiac arrest, our patient resumed budesonide treatment and almost normalized his diarrhea without affecting his serum potassium levels. Following successful resuscitation, no other cause of cardiac arrest could be proven.

Survival from out-of-hospital cardiac arrest is poor [9]. The interventions linking a patient with cardiac arrest to survival with a good cerebral outcome are known as the chain of survival. This includes early recognition of cardiac arrest, early bystander CPR, early defibrillation and early advanced life support including effective post-resuscitation care. Effective chest compressions with correct chest compression depth and frequency are crucial for a successful outcome [9].

This case shows the importance of correcting an underlying cause of cardiac arrest and that survival with a good neurological outcome is possible despite prolonged cardiac arrest.

## Conclusion

Lymphocytic colitis with diarrhea may lead to severe hypokalemia and, ultimately, cardiac arrest. Monitoring of electrolyte levels should be considered in patients with microscopic colitis and diarrhea, especially during changes in symptoms and treatment. During cardiac arrest, the treatment of reversible causes is essential. Resuscitation should generally be continued as long as a shockable rhythm persists. This case demonstrates that survival with a favorable neurological outcome is possible despite prolonged cardiac arrest.

## Consent

Written informed consent was obtained from the patient for publication of this case report and any accompanying images. A copy of the written consent is available for review by the Editor-in-Chief of this journal.

## Competing interests

The authors declare that they have no competing interests.

## Authors' contributions

KAG was the main writing author. JK contributed with expert knowledge on lymphocytic colitis and proofread the report. BL was a co-writer, contributed with expert knowledge on cardiac arrest and proofread the report. All authors read and approved the final manuscript.
